# Arthroscopic management for early-stage tuberculosis of the ankle

**DOI:** 10.1186/s13018-018-1048-y

**Published:** 2019-01-22

**Authors:** Xiaojun Duan, Liu Yang

**Affiliations:** Centre for Joint Surgery, Southwest Hospital, Third Military Medical University (Army Medical University), Chongqing, 400083 China

**Keywords:** Ankle, Tuberculosis, Arthroscopy, Treatment

## Abstract

**Background:**

Due to atypical clinical presentation, wide use of antibiotics, and lack of specificity in diagnosis, diagnosis of tubercular (TB) infection in joints is increasingly difficult, and misdiagnosis is common. The use of arthroscopy for the diagnosis and treatment of early-stage ankle TB has rarely been reported. This case series intended to present the clinical outcomes of arthroscopic management for early-stage ankle TB.

**Methods:**

Fifteen patients with chronic synovitis of the ankle and suspicious cause of early-stage ankle TB underwent arthroscopic treatment from April 1, 2010, to March 31, 2016. These cases all failed to confirm diagnosis of TB by ankle arthrocentesis. They included seven males and eight females with an average age of 37.5 (8 to 70) in the study. Among them, five cases had history of pulmonary tuberculosis, and six had history of trauma. The procedure included synovial membrane biopsy and debridement. The diagnosis was confirmed by pathologic examination and culture. The treatment was combined with systemic anti-tuberculous drugs. Follow-up measurements included VAS score, AOFAS score, ESR, CRP, and MRI.

**Results:**

After arthroscopic management, 13 cases confirmed TB by pathologic examination and culture, and two cases still remained clinically suspected TB; the rate of confirmed case was 87%. The incision healed well in all cases, and no serious complications were observed. There were significant differences in VAS scores, AOFAS scores, ESR, and CRP between before and after treatment (*P* < 0.01). Joint swelling disappeared or was relieved after 2 months in most patients. Ankle swelling and pain in one patient was improved after changing anti-tuberculous drugs. MRI suggested that all patients had effusion in the articular cavity, accompanied by bone edema of the distal tibia and talus before the treatment. After the surgery, the effusion was significantly reduced, and the signal of bone edema almost disappeared. No recurrent TB was found during the follow-ups.

**Conclusion:**

Arthroscopic management for early-stage ankle TB is minimally invasive, safe, and reliable. It can easily obtain samples from specific area of TB for further confirmation of the diagnosis, while the debridement can also assist in local disease control. For cases of highly suspicious joint TB, arthroscopic biopsy and debridement after transient anti-TB treatment is recommended.

**Level of evidence:**

Level IV, therapeutic case series

**Electronic supplementary material:**

The online version of this article (10.1186/s13018-018-1048-y) contains supplementary material, which is available to authorized users.

## Background

According to WHO’s 2014 estimation, about 9.6 million people in the world were infected with tuberculosis (TB), resulting in 1.5 million deaths, which made TB the primary cause of infectious diseases [1]. In 2015, the annual incidence of tuberculosis in China was 68/100,000. Joint TB accounts for about 3% of the extrapulmonary TB. Occasionally, there would be cases of ankle TB [2–5]. China has shown a high incidence of TB [1, 6], and the problem is becoming even more severe with the emergence of multidrug-resistant TB in recent years [7]. Sinus tract is often concomitant in ankle TB, which would severely affect the motor function of the ankle. The diagnosis of tuberculous infection in a joint is difficult, and misdiagnosis is common due to its atypical clinical presentation, wide use of antibiotics, and lack of specificity in diagnosis.

A few decades ago, ankle arthroscopy was considered unavailable because the joint spaces were too narrow to operate. But in the recent 20 years, it has made astounding advances and been successfully used for osteochondral lesions of the talus, impingement syndrome, ankle arthrodesis, etc. [8–17]. However, there are very few reports on ankle TB treatment [2, 8–12], and arthroscopic management for early-stage ankle TB is even less reported.

The main manifestation of early-stage ankle TB is chronic synovitis. Even though ankle arthrocentesis, bacterial culture, and pathological examination have been performed, it is still difficult to confirm the diagnosis. The authors have been using arthroscopy in treating ankle TB for some years and have achieved satisfying results. In order to further improve the level of diagnosis and treatment, we have followed the clinical outcomes after arthroscopic management for early-stage ankle TB and reported as follows.

## Methods

The inclusion criteria were as follows: (1) patients with chronic ankle synovitis, where inflammatory and traumatic origins have been ruled out, and a strong TB suspicion was present but no infectious origin could be determined; and (2) patients who were able to take oral anti-TB drugs on schedule. The exclusion criteria were as follows: (1) patients who had been diagnosed with ankle TB, preferred conservative treatment, and did not need to obtain samples via arthroscopy; (2) patients who had sinus, sequestrum, and bone destruction; other surgical procedures were often necessary in addition to debridement; (3) patients who had unstable vital signs and were unsuitable for surgery; (4) patients who had low compliance of anti-TB drugs; and (5) patients who had active pulmonary TB, which was contagious and should be transferred to a specialist hospital.

Retrospective study was performed to analyze the patients from April 1, 2010, to March 31, 2016. Fifteen cases were included (seven men and eight women; mean age 37.5 years, range 8–70 years; mean duration of illness 16 months, range 3–36 months). All 15 cases involved unilateral ankle joints, which were swollen and painful. Among them, five cases had history of pulmonary tuberculosis, and six had history of trauma (Table 1). They experienced ineffective long-term oral treatment of non-specific anti-inflammatory drugs. Systemic symptoms of TB such as fever, night sweats, and wasting away were not obvious when they transferred to our hospital. Each patient was diagnosed by detailed clinical, radiological, and laboratory evaluations before surgery. All patients had elevated erythrocyte sedimentation rate (ESR) and C-reactive protein (CRP) levels preoperatively. The primary diagnosis was chronic synovitis of the ankle. Purulent infection, rheumatoid arthritis, traumatic arthritis, etc. were preliminarily ruled out, and we strongly suspected that the cause was TB. These patients had indications for an ankle lesion biopsy. To reduce complications, all patients received anti-TB drug therapy for at least 2–3 weeks, until the ESR level decreased or became normal. The drugs used were isoniazid (300–450 mg/day for adults; 10 mg/kg/day for children), rifampicin (450–750 mg/day for adults; 10 mg/kg/day for children), ethambutol (15–25 mg/kg/day for adults and children), and pyrazinamide (20–25 mg/kg/day). VAS scores and AOFAS scores were assessed prior to the treatment [18]. Ankle anteroposterior and lateral radiographs and MRI were used to determine the extent and area of local disease damage.

**Table 1:** General data of patients

Items	Patients (*n* = 15)
Sex (n)	
Male	7
Female	8
Age (yr)	37.5 (8–70)
Side (n)	
Right	6
Left	9
History of pulmonary tuberculosis (n, %)	5 (33.3%)
History of trauma (n, %)	6 (40.0%)
Time from symptom to surgery (months)	16 (3–36)

### Surgical purpose and technique

The purposes of the surgery included two aspects: (1) for the suspected cases, to directly observe the lesion under arthroscope and to make relevant examinations with the focal tissue (pathological examination, L-J medium culture, TB-PCR) for further confirmation, as well as to improve the treatment plan with more accurate judgments on the prognosis; (2) arthroscopic debridement, i.e., direct removal of necrotic tissue, which could improve the effective concentration of anti-TB drugs in the focal tissue and be an adjunct to local disease control. During the surgery, if the area of articular cartilage damage is less than 2 cm^2^ and the residual cartilage is stable, it is often effective to perform debridement rather than arthrodesis.

Epidural anesthesia or nerve block anesthesia was applied. Patients were placed in supine position, buttocks padded high, and the ankle was maintained in the neutral position. Marker pen was used to mark the superficial peroneal nerve and dorsalis pedis artery. Pneumatic tourniquet was applied with the pressure of 250–300 mmHg. The non-invasive ankle traction was used with the posterior malleolus hanging, which was easy for debridement [13]. Ankle arthrocentesis was performed to drain the joint effusion, and sample of the joint effusion was obtained for acid-fast staining test; saline 10–20 mL was injected into the articular cavity. After incision, a 30° ankle arthroscope (2.7 mm in diameter, Smith & Nephew Endoscopy, Andover, MA) was used. Ankle exploration was carefully performed through the anteromedial incision between the anterior tibialis tendon and the medial malleolus [19]; further exploration was conducted through the anterolateral incision at the lateral of the peroneus tertius tendon under arthroscope. In general, synovial fluid that is purulent and turbid could be seen under arthroscope, and sometimes fibrous protein and necrotic tissue, hyperplasia of synovial tissue, congestion, and pale areas could also be seen. The diversity of the cartilage damage could be manifested as cartilage degeneration, defects, layering, and subchondral bone exposure. Next, typical pathological tissue and necrotic tissue were taken under the arthroscope as samples for pathological examination, Lowenstein-Jensen medium culture, TB-PCR, etc. Shaver, basket forceps, pituitary rongeur, and other instruments were used to completely remove the necrotic tissue and degenerative synovia of the articular cavity. A drainage tube was placed to infuse 0.1 g isoniazid into the joint cavity and then clamped. The negative pressure drainage could be started 12 h after surgery. The incision was closed with 3-0 sutures. After surgery, all patients were given plaster casts to keep the ankle in the functional position.

Postoperative management for ankle TB is very important. Patients with fever or pain should receive symptomatic treatment. The volume of the negative pressure drainage must be observed carefully, and drain removal is usually performed 24–48 h after surgery. In addition, patients are encouraged to have high-protein diet to improve nutrition. It is an important principle to reduce the movement of ankle joint or to conduct moderate exercise without ankle pain. Weight-bearing exercise could be done 2 weeks postoperatively when the sutures are taken out. Postoperative anti-TB drugs are also very important, with the principles of combined, standardized, adequate, and full course. The quadruple therapy of isoniazid, rifampicin, pyrazinamide, and ethambutol was continued for 3 months, triple therapy of ibuprofen, rifampicin, and pyrazinamide for another 3 months, and then the combination of isoniazid and rifampicin; the total oral administration of anti-TB drugs lasted up to 18 months. The patients could walk with gradual weight-bearing after the sutures were taken out 2 weeks after surgery.

### Follow-up and statistical analysis

The patients were followed up at postoperative 2 and 6 weeks and 3, 6, 12, 18, and 24 months. The symptom changes and the laboratory tests such as ESR, CRP, blood routine, liver and kidney function were re-checked. VAS and AOFAS scores were checked at the last follow-up. The cure criteria were as follows: after 3 months of drug discontinuance, patients could be discharged when they could walk painlessly for 1 km and ESR was normal for more than 3 months postoperatively. The enumeration data was expressed with mean ± standard deviation; statistical analysis was conducted by paired *t* test with SPSS 16.0; *P* < 0.05 indicated statistical significance.

## Results

The diagnosis of 13 patients was confirmed postoperatively by pathologic analysis and culture of mycobacterium tuberculosis. The remaining two cases still failed to be diagnosed by biopsy. Therefore, with the numbers available in this study, through pathological examination and bacterial culture of the samples obtained from arthroscopy, 87% of the total cases that could not be diagnosed by ankle arthrocentesis preoperatively could be confirmed. We continued giving all 15 cases anti-TB drugs after arthroscopic management, and their symptoms disappeared completely. All incisions were healed well. There were significant differences in ESR, VAS, and AOFAS scores before and after treatment (Table 2). No severe complications were observed, and all patients were cured. MRI examination suggested that all patients had preoperative effusion in the ankle articular cavity, accompanied by bone edema of the distal radius and the talus. After treatment, the effusion was significantly reduced, and the bone edema signal almost disappeared. According to the AOFAS score in the last follow-up, nine cases were excellent, three cases good, and three cases acceptable.

**Table 2:** Comparison of results before treatment and at the last follow-up

	VAS score	AOFAS score	ESR (mm/h)	CRP (mg/L)
Before treatment	7.1 ± 2.2	38 ± 12	57 ± 24	28 ± 12
At the last follow-up	1.0 ± 0.5	85 ± 7	15 ± 4	5 ± 2
Paired *t*-test	*P* < 0.01	*P* < 0.01	*P* < 0.01	*P* < 0.01

In one case, the symptoms were not significantly improved 3 months postoperatively. After consulting with pharmacologists, anti-TB drugs were adjusted and the patient was cured in later follow-ups. We chose three typical cases as follows.

### Case 1

**Fig. 1:**
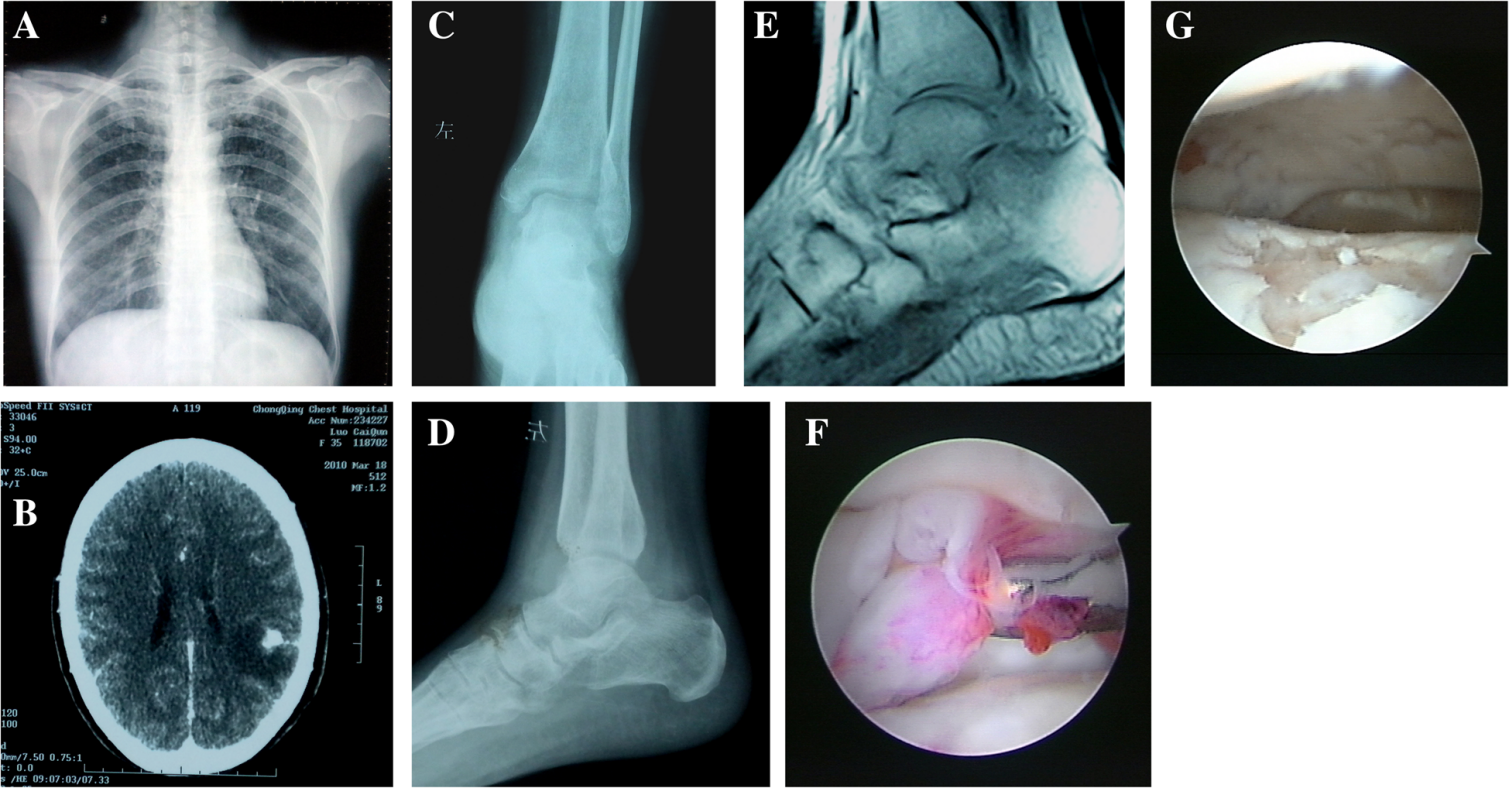
**a** Chest radiograph suggesting tuberculosis. **b** Cranium CT showing intracranial lesions. **c** Preoperative radiograph suggesting ankle diffuse osteoporosis (anteroposterior view). **d** Preoperative radiograph suggesting ankle diffuse osteoporosis (lateral view). **e** Preoperative MRI suggesting inflammatory tissue of ankle and subtalar joint. **f** Hyperplasia of synovial tissue under ankle arthroscope. **g** Cartilage defects can be seen after debridement, with the residual cartilage remaining stable


A female, 36 years old, had symptoms of pulmonary TB and intracranial TB which were alleviated after using anti-TB drugs, but the swelling and pain of the left ankle lasted for 1 year. Ankle TB was confirmed with the pathological examination of focal tissue after arthroscopy. It showed hyperplasia of synovial tissue, pale areas, regional congestion, scattered fibrous protein, and necrotic tissue, but the residual cartilage remained stable. Arthroscopic debridement was performed to remove the ankle scarring tissue and improve the motor function of the ankle. Re-examination showed she was cured with an AOFAS score of 93 points (Fig. 1, Additional file 1: Video S1).

Additional file 1:Video S1. Ankle arthroscopy to debride tuberculous infection. (MP4 2170 kb)


### Case 2

**Fig. 2:**
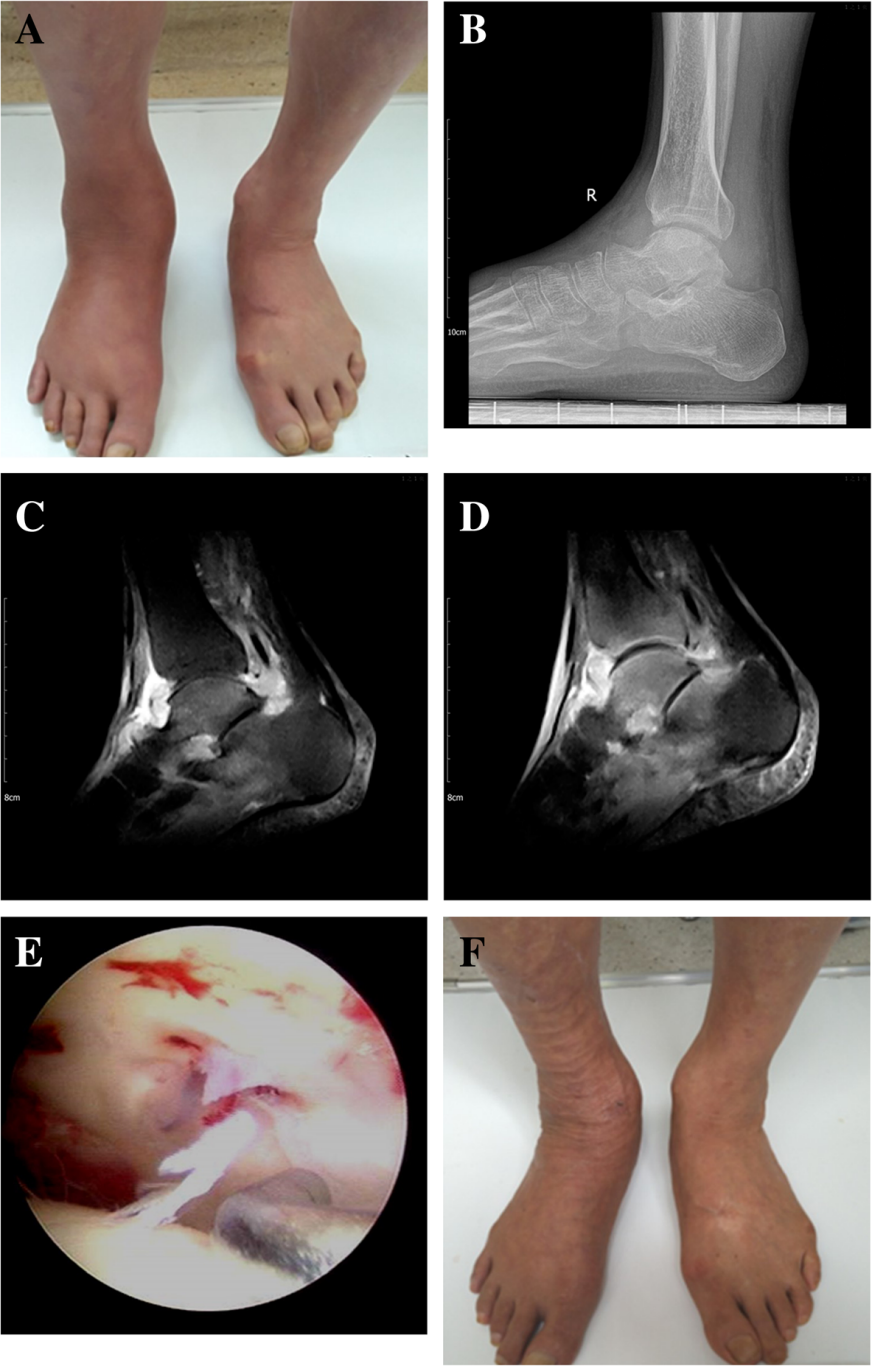
**a** Preoperative right ankle swelling. **b** Preoperative radiograph suggesting no bone destruction in the ankle. **c** Ankle MRI before rheumatoid arthritis treatment, suggesting ankle effusion and mild bone edema of the talus. **d** Re-examination of MRI after rheumatoid arthritis treatment, suggesting increased edema of the talus and formation of joint cavity lesions. **e** Small area of cartilage damage under arthroscope. **f** Right ankle swelling improved 6 weeks postoperatively

The right ankle of a 64-year-old male gradually became swollen and painful. The results of venous blood test for rheumatoid factor (RF), anti-cyclic citrullinated peptide (CCP) antibody, anti-keratin antibody (AKA), and HLA-B27 were all negative. The patient received treatment for rheumatoid arthritis and was given analgesic drugs orally; no hormone was taken orally. The curative effect was poor. After 6 months of treatment, the ankle joint pain and swelling were aggravated and claudication occurred. Venous blood test was performed again, and the results of T-SPOT.TB test and TB antibody were positive; chest radiograph showed pulmonary TB. Ankle arthrocentesis was conducted; the result of bacterial culture was negative and suspected TB for pathological examination. Quadruple anti-TB therapy (isoniazid, rifampicin, pyrazinamide, ethambutol) was given orally. After 3 weeks of treatment, the swelling of the right ankle joint was relieved. ESR was improved from 42 mm/h before treatment to 25 mm/h. Then ankle arthroscopy was performed. Under arthroscope, a small area of defect in the ankle cartilage was seen, but the cartilage remained stable without looseness; the ankle joint had a large amount of fibrous protein as well as hyperplasia and hyperemia of the synovial tissue. Samples were taken for TB culture and pathological examination, and then ankle debridement was performed. Postoperative pathological examination confirmed ankle TB. Anti-TB treatment was continued for 18 months. At the last follow-up, the symptoms of the ankle joint disappeared, the ESR was 8 mm/h, and the AOFAS score improved from 49 points before treatment to 94 (Fig. 2).

### Case 3

**Fig. 3:**
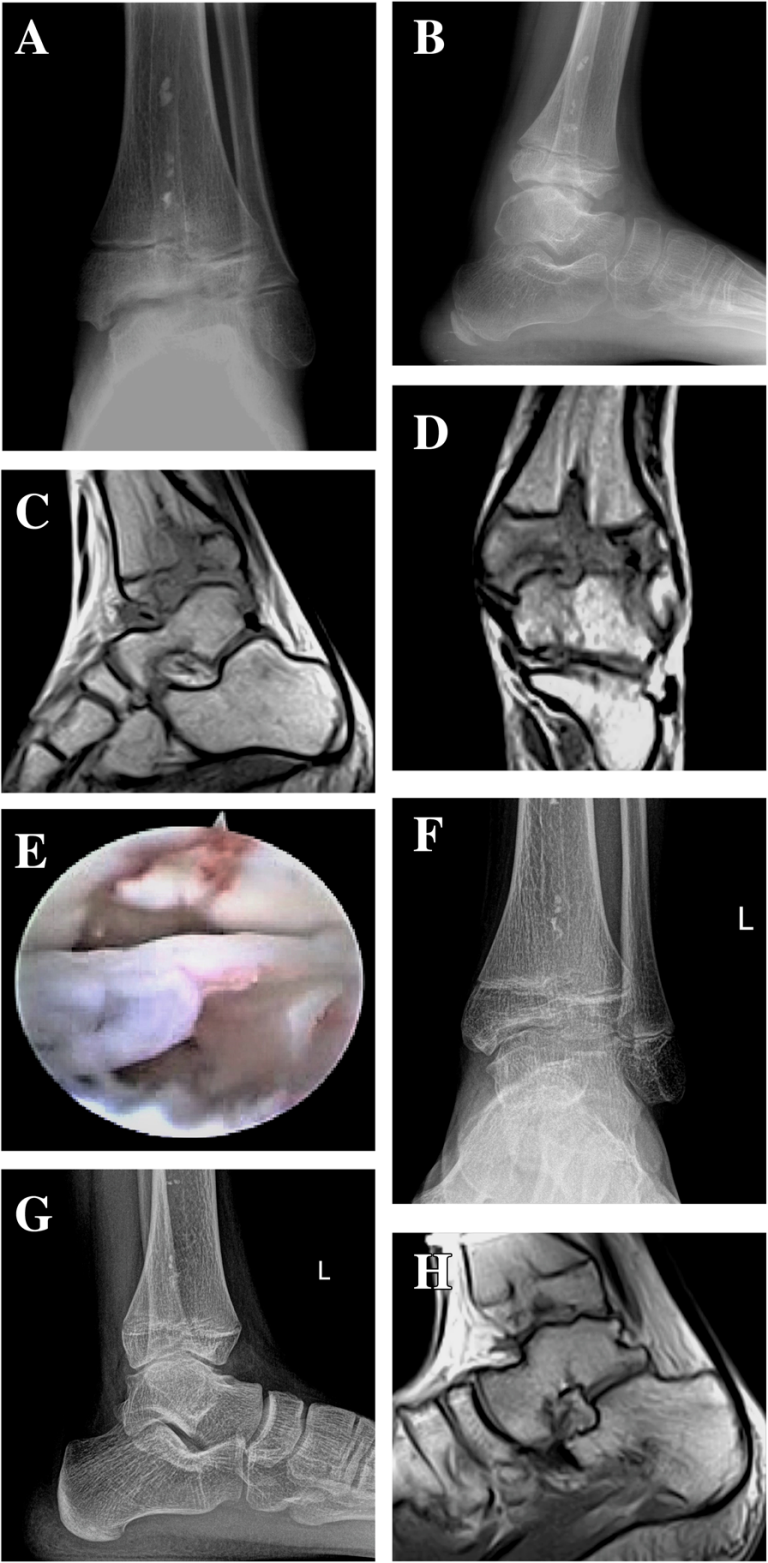
**a** Preoperative radiograph suggesting unsmooth ankle joint space and epiphyseal injury of the tibia (anteroposterior view). **b** Preoperative radiograph suggesting unsmooth ankle joint space (lateral view). **c** Preoperative MRI suggesting ankle joint damage and hyperplasia of synovial tissue (sagittal view). **d** Preoperative MRI suggesting ankle joint damage and hyperplasia of synovial tissue (coronal view). **e** Articular cartilage defects, massive synovial hyperplasia, and scar tissue formation seen during arthroscopy. **f** Radiograph 5 years postoperatively suggesting that the ankle joint is not smooth, but the epiphyseal injury of the tibia is better than before surgery (anteroposterior view). **g** Radiograph 5 years postoperatively suggesting that the ankle joint is not smooth but is better than before surgery (lateral view). **h** MRI 5 years postoperatively suggesting that the ankle joint is not smooth (sagittal view)


A male, 11 years old, suffered from pain in the left ankle joint after trauma and limitation of motion for 10 years, and the symptoms aggravated with claudication for 1 year. Physical examination showed swelling of the left ankle joint, extensive tenderness, normal skin temperature, and limited range of motion for ankle plantar flexion and dorsi flexion. Venous blood test showed no abnormalities in ESR, CRP, and blood routine. Radiograph and MRI suggested hyperplasia of synovial tissue in the articular cavity and epiphyseal injury of the distal tibia. Ankle arthrocentesis was conducted, and a small amount of turbid liquid was drained. No diagnosis of ankle TB was suggested. Preoperative diagnoses were (1) traumatic ankle synovitis and (2) epiphyseal injury of the left distal tibia. We initially planned to perform articular cavity debridement; however, when we conducted the surgery, we saw obvious hyperplasia of synovial tissue and the cartilage was obviously damaged; TB was highly suspected. Considering that the patient was still a child, ankle arthrodesis was not suitable in this case. Therefore, we only performed articular cavity debridement after obtaining the tissue sample for biopsy. Pathological examination confirmed ankle TB, and the result was also positive for TB-PCR. The patient was prevented from weight bearing for 6 weeks postoperatively; anti-TB treatment of rifampicin, isoniazid, and pyrazinamide were given orally; his nutrition was strengthened. Regular follow-ups were conducted. The swelling of the posterior malleolus was gradually relieved. After the reexamination at 6 months postoperatively, his anti-TB therapy was adjusted and only rifampicin and isoniazid were continued for maintenance treatment of 12 months. At the last follow-up at 5 years postoperatively, the patient’s left ankle swelling and pain disappeared, and the range of motion for ankle plantar flexion and dorsi flexion was basically normal. The AOFAS score improved from 57 points preoperatively to 97, with ESR 1 mm/h. Radiograph and MRI suggested that the ankle joint space was slightly narrow, the surface of tibiotalar joint was not smooth, and the lesion of synovial hyperplasia disappears. The patient was satisfied with the results (Fig. 3, Additional file 2: Video S2 and Additional file 3: Video S3).

Additional file 2:Video S2. Claudication gait before surgery. (WMV 19928 kb)



Additional file 3:Video S3. Gait is normal 5?years postoperatively. (WMV 21347 kb)


## Discussion

Tuberculosis, which is mainly found in developing countries, still remains as a major global health problem for 20 years since WHO announced it a global public health issue [1, 2, 20]. In 2010, China published an official guideline for the diagnosis and treatment of pulmonary TB. However, there are no guidelines for joint TB, whose treatment is still referred to guidelines for pulmonary TB [4]. Due to its atypical symptoms, joint TB is often neglected and misdiagnosed [21–23]. According to references [9, 24–26], some patients were given open surgery while receiving anti-TB drugs, but this method would be more traumatic and cause difficulty in postoperative incision healing. Arthroscopy has made great progress in many countries. Yet arthroscopy for ankle TB is still rarely reported, and the effectiveness and safety of the technique is unclear.

Early diagnosis and treatment of joint TB has a decisive significance on the prognosis [2, 27–33]. MRI examination is helpful in the early diagnosis of ankle TB [34]. It can show clear vision of effusion in the articular cavity and sometimes with low-signal focal tissue, and high signal of edema is seen in the distal tibia and the talus (kissing sign). Under MRI, the signal dispersion range of TB is usually greater than osteoarthritis. It is often confined to the lateral of the tibiotalar joint rather than bilateral, which is seen in the case of tumor or early bone necrosis. It is also easy to observe the soft tissue abscess near the ankle and even in the subtalar joint by MRI. Due to its non-invasiveness and sensitiveness, MRI is used as a routine examination of ankle TB. Arthroscopy is of great diagnostic value for cases that cannot be confirmed by ankle arthrocentesis. Through this minimally invasive surgery, typical lesion samples can be obtained for pathological examination, and the positive rate was higher than that by arthrocentesis; besides, the sample volume obtained by arthroscopy was also more, which was very useful for bacterial culture and other related examinations. Arthroscopy can also timely correct misdiagnosed cases, such as in our case 3. Cases that cannot be confirmed before surgery are often quite intractable. In this study, the rate of confirmed cases after arthroscopy is 87%, which we believe is very impressive.

Arthroscopy could not only provide samples for pathological examination and L-J medium culture to confirm the diagnosis, but also be an adjunct to local disease control. The purposes of surgical treatment are first, to further confirm the diagnosis, especially for early joint TB; and second, the removal of necrotic tissue, fibrous protein, and hyperplasia of synovial tissue, which would be conducive to reducing the inflammatory response [35–37]. When the TB gradually stabilizes, the debridement of hypertrophic scarring tissue could further improve the ankle function. There are only a small number of follow-up cases reported for the surgical technique of ankle TB [38–46]. Due to the lack of large sample studies, it is difficult to decide the optimal surgical approach. Traditionally, the surgical treatment for advanced active joint TB includes debridement, arthrodesis, and arthroplasty combined with a certain period of anti-TB therapy [36]. Of course, intraoperative exploration of articular cartilage damage is the most reliable evidence for determining which surgical procedure to perform. Small area of cartilage damage, especially when the residual cartilage is stable, such as in our case 1, could consider using the arthroscopic debridement to retain ankle function; results show that the patient’s prognosis is satisfactory. In large areas of cartilage damage, such as in our case 3, arthroscopic debridement and effective anti-TB drugs will be enough for children to achieve satisfactory prognosis; ankle arthrodesis is not necessary.

Ankle TB is very rare and published data of arthroscopic management for it is rather limited. This study also has some limitations, such as small sample size and relatively short follow-up period. At the same time, due to limited number of cases, it is difficult to conduct comparative studies between arthroscopy and pure drug therapy. Thus, the therapeutic value of arthroscopy is also difficult to determine. Future multi-center controlled studies are expected to address these limitations.

## Conclusion

Arthroscopic management for ankle TB is minimally invasive, safe, and reliable. It can easily obtain samples for further confirmation, and the removal of lesions may be an adjunct to local disease control. Arthroscopy can also help evaluating cartilage damage, providing prognostic information of ankle joint function. During the surgery, if the area of articular cartilage damage is less than 2 cm^2^ and the residual cartilage is stable, it is often effective to perform debridement rather than arthrodesis.

## Abbreviations

AOFAS: American Orthopedic Foot and Ankle Society
ESR: Erythrocyte sedimentation rate
HLA: Human leukocyte antigen
MRI: Magnetic resonance imaging
VAS: Visual analogue scale
WHO: World Health Organization
